# The Contribution of Muscle Innate Immunity to Uremic Cachexia

**DOI:** 10.3390/nu15132832

**Published:** 2023-06-21

**Authors:** Pasquale Esposito, Daniela Verzola, Michela Saio, Daniela Picciotto, Marco Frascio, Alessandro Laudon, Valentina Zanetti, Giuliano Brunori, Giacomo Garibotto, Francesca Viazzi

**Affiliations:** 1 Division of Nephrology, Dialysis and Transplantation, IRCCS Ospedale Policlinico San Martino, 16132 Genova, Italy; pasquale.esposito@unige.it (P.E.); michela.saio@virgilio.it (M.S.); daniela.picciotto@hsanmartino.it (D.P.); valentinazanetti94@gmail.com (V.Z.); francesca.viazzi@unige.it (F.V.); 2 Department of Internal Medicine, University of Genova, 16132 Genova, Italy; daverz@libero.it; 3 Division of Surgery, IRCCS Ospedale Policlinico San Martino, 16132 Genova, Italy; marco.frascio@unige.it; 4 Department of Surgical Sciences and Integrated Diagnostics, University of Genova, 16132 Genova, Italy; 5 Division of Nephrology, Ospedale Santa Chiara, 38122 Trento, Italy; alessandro.laudon@apss.tn.it (A.L.); giuliano.brunori@apss.tn.it (G.B.)

**Keywords:** innate immunity, muscle, CKD, protein metabolism, amino acids

## Abstract

Protein energy wasting (PEW) is a common complication both in chronic kidney disease (CKD) and end-stage kidney disease (ESKD). Of note, PEW is one of the stronger predictors of morbidity and mortality in this patient population. The pathogenesis of PEW involves several mechanisms, including anorexia, insulin resistance, acidosis and low-grade inflammation. In addition, “sterile” muscle inflammation contributes to PEW at an advanced CKD stage. Both immune and resident muscle cells can activate innate immunity; thus, they have critical roles in triggering “sterile” tissue inflammation. Toll-like receptor 4 (TLR4) can detect endogenous danger-associated molecular patterns generated or retained in blood in uremia and induce a sterile muscle inflammatory response via NF-κB in myocytes. In addition, TLR4, though the activation of the NLRP3 inflammasome, links the sensing of metabolic uremic stress in muscle to the activation of pro-inflammatory cascades, which lead to the production of IL-1β and IL-18. Finally, uremia-induced accelerated cell senescence is associated with a secretory phenotype that favors fibrosis in muscle. Targeting these innate immune pathways could lead to novel therapies for CKD-related PEW.

## 1. Introduction

Protein energy wasting (PEW) is a common complication across trajectories of chronic kidney disease (CKD) and end-stage kidney disease (ESKD) [1]. Of note, PEW is one of the stronger predictors of morbidity and mortality in this patient population [2,3,4]. The pathogenesis of PEW is complex and involves a multitude of different mechanisms, including anorexia, insulin resistance and acidosis [5]. In addition, inflammation has a crucial role in the development and progression of wasting [5]. In patients with ESKD, C-reactive protein and interleukin-6 (IL-6) increase over time, and serum albumin and body mass index decrease, especially in elderly subjects, in the male gender and in diabetic subjects [6]. While, in the past, skeletal muscle was considered an innocent victim of inflammation, recent acquisitions have shown that muscle plays an active role in the innate immunity response, which is linked to the muscle protein metabolism. In this review, we discuss the role of innate immunity in skeletal muscle as a mediator of CKD-related PEW. In particular, we highlight recent advances in the role of Toll-like receptor 4 (TLR4) and NOD-, LRR- and pyrin-domain-containing protein 3 (NLRP3) inflammasomes in muscle, and we discuss the possible benefits and limitations of targeting the inflammatory response in the treatment of CKD-related PEW.

## 2. Inflammation in CKD

Chronic low-grade inflammation, mainly through the IL-6 and the NLRP3 inflammasome signaling pathways, increases the risk of developing anemia, accelerated atherosclerosis, insulin resistance, diabetes mellitus and osteoporosis in CKD [7,8]. As shown by early studies, acute-phase response biomarkers predicted future cardiovascular events in healthy subjects and in patients with unstable angina [9]. More recently, similar results were obtained in patients with CKD [10], a patient category with an extremely elevated risk of cardiovascular complications. Thus, a current hypothesis is that non-traditional risk factors, such as oxidative stress, inflammation and PEW, defined together in the malnutrition–inflammation–atherosclerosis (MIA) or malnutrition–inflammation complex syndrome (MICS) [11,12], may determine premature vascular senescence and tissue damage in CKD patients. Of note is that in patients with CKD, higher levels of high-sensitivity C-reactive protein (hsCRP), IL-6, TNF-alpha and other cytokines have been shown to be associated with a higher risk of all-cause and cardiovascular mortality [13,14,15,16,17].

Inflammation activates specific catabolic or anti-anabolic pathways in uremic muscle [17,18]; both whole body and peripheral tissue net protein breakdown is accelerated in inflamed patients under renal replacement therapy [19,20]. Despite major steps forward, our understanding of uremic pathophysiology, the etiology and the mediators of CKD-related sterile muscle inflammation is still only partially understood.

## 3. Mechanisms and Mediators Contributing to Inflammation in Patients with CKD

In addition to alterations in the intestinal microbiome and dysbiosis, a number of molecules may cause inflammation in patients with CKD. These include CKD-related factors, such as modified lipoproteins, adipocyte alterations, premature ageing, oxidative stress, metabolic acidosis, mitochondrial dysfunction, components of the renin–angiotensin system and alterations of the calcium–phosphate metabolism [21]. Additionally, metabolic signals associated with obesity can cause mesangial hypertrophic glomerulonephritis, which can lead to progressive focal segmental glomerulosclerosis with severe inflammation–fibrosis–atherosclerosis [21]. In addition, lifestyle habits, comorbidities and aging are adverse factors that may overlap with CKD-related factors [22,23]. Inflammation may also be maintained through a reduced cytokine elimination due to a decreased glomerular filtration rate and kidney metabolic activity [24]. Mechanisms and mediators contributing to inflammation in CKD have been recently reviewed [20].

## 4. The Innate Immune System in Muscle

**Figure 1:**
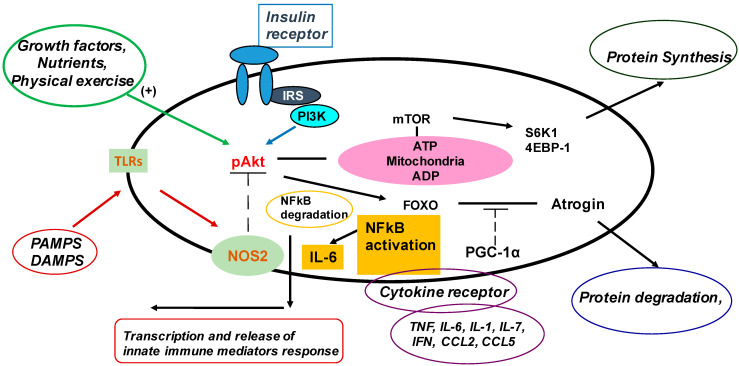
The innate immune response in skeletal muscle. Skeletal muscle has an afferent sensory limb containing, among others, receptors for immune modulators, such as Toll-like receptor (TLR) agonists, damage/pathogen-associated molecular patterns (D/PAMPs), cytokines and chemokines (IL, IFN and TNF) and growth factors and hormones (e.g., insulin, growth hormone, catecholamines, etc.). Upon TLR activation, muscles respond by activating NF-κB and other transcription factors to produce and release innate immune factors (efferent limb). The muscle innate immune response to pathogens and DAMPs can exert profound effects on muscle protein metabolism due to an imbalance between protein synthesis and an increase in rates of protein degradation, mediated by up-regulation of the ubiquitin–proteasome, autophagy or calpain. In addition, inflammatory cytokines cause resistance to anabolic signals by antagonizing the actions of anabolic hormones and exercise-induced signals. While an increased release of amino acids from skeletal muscle is of major benefit to support glucose and acute-phase protein synthesis, on a long-term basis, the inflammatory response is followed by cachexia and has been associated with worse outcomes. Abbreviations used in the figure are defined in the text. Modified from: R. A. Frost, C. H. Lang Regulation of muscle growth by pathogen-associated molecules. *J. Anim. Sci.* **2008**, *86*, E84–E93 [28].

There is an increasing understanding of the role played by skeletal muscle in the immune response and inflammation, mainly on the activation of the innate immune system in muscle [24,25]. According to an emergent hypothesis, skeletal muscle is now regarded as a component of the innate immune system, with primary importance in the response to bacterial infection, as summarized in Figure 1.

In this model [26,27,28], the afferent limb is composed of a number of receptors that recognize several pathogen-associated molecular patterns (PAMPs) and damage-associated molecular patterns (DAMPs), while the efferent limb is composed of mediators produced by muscle cells acting either in an autocrine/paracrine and/or endocrine fashion [28].

Pattern recognition receptors (PRRs) expressed in muscle are able to sense a number of molecules (such as RNA, DNA or microbial cell wall components), which are derived from invading pathogens or the intestinal microbiota [26,27]. In addition, several intermediate products of metabolism (e.g., free fatty acids, advanced glycation end products, mitochondrial DNA, etc.) called metabolism-associated molecular patterns (MAMPS) are capable of activating PRRs [26]. Toll-like receptors (TLRs), which are a major component of the native immunity afferent limb (Figure 1) [28], can activate transcription factors that up-regulate the expression of pro-inflammatory cytokines in several cell types and tissues, including skeletal muscle [25,26,27,28]. Both TLRs’ functions and signaling are dependent on their location, with receptors located on the cell surface (i.e., TLR1, TLR2, TLR4, TLR5, TLR6 and TLR10) usually recognizing pathogenic components, such as proteins (LPS and flagellin) or lipids (mainly lipoproteins). The remaining TLRs are localized to the endosomal compartment. Comprehensive and detailed listings of common PRRs in innate immunity have recently been published [27,28].

Cytokine receptors, which recognize pro-inflammatory cytokines that circulate in extra-cellular fluids, are another important component of the afferent limb [28]. Skeletal muscle expresses receptors for IL-6, TNF-α and IL-1, and their activation is followed by a number of metabolic responses [25,28].

TLRs are not the only major PRRs in the host defense mechanism expressed in skeletal muscle. Pathogenic microorganisms that escape initial detection can be present intra-cellularly and can be recognized by specialized NLRs. For example, nucleotide-binding and oligomerization domain (NOD)-like receptors (NLRs), which serve as intra-cellular pathogen sensors in immune tissues, are also found in muscle [25]. NOD1 and NOD2 detect bacteria-specific peptidoglycans (γ-d-glu-meso-diaminopimelic acid and muramyl dipeptide (MDP), respectively), resulting in the up-regulation of pro-inflammatory signaling pathways via the activation of the nuclear factor κ light-chain enhancer of activated B cells (NF-κB) [26,27].

## 5. Effects of Innate Immunity on Muscle Protein Metabolism

The muscle innate immune response to pathogens and DAMPs can cause important changes in muscle protein metabolism. Early studies have demonstrated a decrease in lean body mass (LBM) and an increase in whole-body nitrogen excretion in patients with long-standing sepsis and inflammation [29,30]. This reduction in LBM results from an imbalance between decreased protein synthesis and an increase in the rate of protein degradation mediated by the up-regulation of ubiquitin–proteasome, autophagy or calpain [25]. IL-1β activates NF-κβ signaling and increases the expression of IL-6 and atrogin-1 in C2C12 myocytes [30]. The intra-cerebroventricular injection of IL-1β caused muscle wasting in mice [30]. In addition, inflammatory cytokines cause resistance to anabolic signals by antagonizing the actions of insulin/IGF-I and exercise-induced signals [31]. Functionally, an increased release of amino acids from skeletal muscle is of major benefit in supporting glucose and acute-phase protein synthesis. However, on a long-term basis, it is followed by wasting and/or cachexia [26,32,33], and has been associated with worse outcomes. Due to muscle mass typically representing a large proportion (60% to 70%) of the total body weight, the muscle innate immunity response constitutes an overlooked and potentially important element of the immune system.

## 6. Patients with CKD5 Have an Activated Canonical TLR4–NF-κB–IL-6 Pathway in Muscle

To evaluate a possible role of TLRs in initiating inflammation in the muscle of CKD patients, we studied the expression profiles of selected TLR4 downward genes in the rectum adbominis muscle of CKD5 patients at the time of the insertion of a peritoneal dialysis catheter [34]. We observed that CKD5 patients had an up-regulation of TLR4 and a downward NF-κB-dependent production of TNF-α and pro-inflammatory cytokines in muscle [33]. To investigate whether uremia-associated circulating factors stimulate TLR4 signaling, we treated C2C12 myotubes with uremic serum. In myotubes, uremic serum induced a marked increase in TLR4 mRNA, an effect that was prevented by specific TLR4 inhibitor. In addition, uremic serum increased TNF-α mRNA expression, up-regulated p38 MAPK phosphorylation and reduced Akt phosphorylation in cultured myotubes.

In muscle, the activation of the Akt signaling pathway mediates the balance between catabolism and anabolism. In animal models of CKD, IGF-1 or insulin resistance, glucocorticoids, acidosis, inflammation or cancer led to alterations in the insulin-stimulated intra-cellular signal and a decrease in phosphorylated *p*-Akt [34,35]. In turn, a low *p*-Akt activated caspase-3 and the ubiquitin–proteasome system (UPS). In addition, a low *p*-Akt leads to the stimulation of the expression of the regulators of protein degradation atrogin-1 and MuRF1. Caspase-3 can also increase proteasome-dependent protein breakdown [35]. As a fingerprint of the decreased pAkt, we also observed an over-expression of atrogin-1 and MuRF1 in the muscle of CKD patients. In addition, we observed that p38 MAPK, a second messenger for TNF-α [36,37,38], was over-expressed in CKD muscle. The p38 MAPK activity in muscle has been shown to be over-expressed in aging [39], diabetes [40], limb immobilization [41] and neurogenic atrophy [42], suggesting that TNF-α/p38 MAPK-driven signaling is a pathway common to many catabolic conditions. In our study, p38 MAPK and PKC inhibitors resulted in a marked decrease in the serum-induced TLR4 mRNA over-expression in cultured myocytes. Collectively, these results showed that TLR4 and its downward canonic inflammatory cascade were activated in the muscle of patients with CKD5, and suggested that enhanced TLR4 signaling contributed to the up-regulation of muscle native immunity.

## 7. A TLR4–NLRP3 Inflammasome Pathway Is Also Activated in Muscle of CKD5 Patients

**Figure 2:**
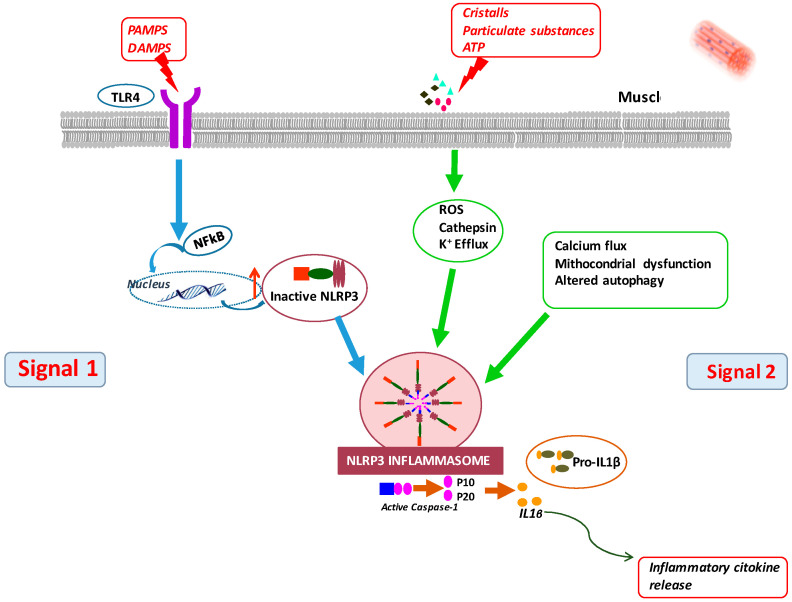
A two-phase process, with an inception and an activation step, leads to the activation of NLRP3 inflammasome. During the inception phase, TLRs or TNF activate nuclear factor kappa-B (NF-κB), up-regulating NLRP3 and IL-1β proteins. During the activation phase, several DAMPs (including uric acid, cholesterol and amyloid β-protein) induce NLRP3 inflammasome assembly and subsequent activation. Following its activation, NLRP3 recruits the adaptor protein ASC through PYD–PYD interactions, polymerizing ASC. Pro-caspase-1 is recruited by ASC and undergoes auto-cleavage into caspase-1, causing the maturation of IL-1β and IL-18 and triggering an immune response. Abbreviations are defined in the text.

The innate immune response can also be triggered due to LPS binding to cytoplasmic PRRs, which are multi-protein complexes referred to as inflammasomes [26]. The most studied is the inflammasome NLRP3, which, in contrast to TLRs, does not function through the activation of NF-κB. For its activation, a complex association is required with specific proteins. The NLRP3 inflammasome is composed of a sensor, NLRP3, an adapter protein, ASC, and an effector enzyme, caspase-1, and it is activated by a number of inducers. Inflammasome activation leads to caspase-1 activation and the release of IL-1β and IL-18, which are critically involved in inflammatory responses [26] (Figure 2).

NLRP3 inflammasome signaling is contained and active in muscle cells [24,43]. Sepsis up-regulates both NLRP3 and IL-1β expression in whole muscle, ultimately leading to elevated concentrations of circulating IL-1β [24]. NLRP3 knock-out mice were protected from several aspects of sepsis and age-related dysfunction [44], while NLRP3 over-expression contributed to “sterile” inflammation and wasting in models of aging [43,44,45], Duchenne-associated muscle atrophy [46] and sepsis [47]. NLRP3 inflammasome activation also plays a role in atherosclerosis [48], frailty and cognitive decline [49], all settings commonly associated with CKD.

Using rectum abdominis biopsy samples, we observed that a TLR4/NLRP3/caspase-1-driven inflammatory response took place in the muscle of non-dialysis-treated CKD5 patients [50]. This response was associated with the up-regulation of Nox 4 and nitrotyrosine, which are both related to enhanced oxidative/nitrative stress. In addition, using C2C12 mouse cells exposed to uremic serum, we observed that several components of NLRP3 inflammasome were quickly activated, leading to IL-1β gene expression, an effect that is prevented through the inhibition of TLR4 signaling [50].

## 8. Clinical Associations of Muscle Inflammation in Patients with CKD5

**Figure 3:**
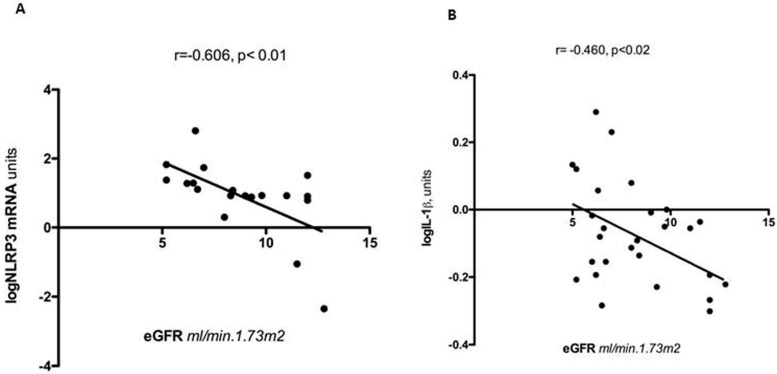
(**A**,**B**). Relationships between muscle NLRP3 mRNA (**A**), logIL-1 β protein (**B**) and estimated glomerular filtration rate (eGFR) in patients with chronic kidney disease. IL-1β, interleukin-1β; NLRP3, NOD, LRR and pyrin-domain-containing protein 3 (Reprinted from Ref. [50]. Verzola D.; et al. *JCSM Rapid Commun.* **2023**, *6*, 50–61. Published online in Wiley Online Library (wileyonlinelibrary.com) https://doi:10.1002/rco2.75. CC-BY-NC license).

To better understand the significance of TLR4-dependent inflammatory pathways over-expressed in uremic muscle, we studied the associations between muscle TLR4 and clinical/laboratory parameters. Both proxies of PEW and the progressive loss of residual renal function are predictive of muscle TLR4 [33]. The TLR4 content in muscle increased progressively along with the progressive decline in residual renal function, with a two-fold increase in muscle TLR4 expression as eGFR declined from 13 to 4 mL/min in the cohort of non-dialysis-treated patients studied. In addition, both muscle NLRP3mRNA and IL-1β, the final products of the inflammasome, were also inversely associated with eGFR [50] (Figure 3, reprinted from Ref. [50]), implying that the inflammasome was progressively activated in the skeletal muscle of CKD5 patients, along with progression towards the uremic stage. Collectively, TLR4 up-regulation in muscle appeared to be a component of the stress response that took place in uremia. The observed changes in innate immunity components could not be accounted for by metabolic acidosis, since patients received bicarbonate supplements. As compared to metabolic acidosis, which is often already observed at an early CKD stage [51], the up-regulation of native immunity in muscle is a late muscle stress response observed in the pre-dialytic stage.

Individual DAMPs that can elicit the NLRP3 inflammasome response in skeletal muscle in uremia are only partially known. Several of the already recognized DAMPs (free cholesterol, uric acid, ROS, uremic toxins, some nucleotides and nucleosides, nuclear and mitochondrial DNA, RNA, DNA-binding molecules, temperature-shock proteins, etc.) can accumulate in blood in uremia [51,52,53,54] and might be responsible for eliciting a tissue inflammatory response. As an example, indoxyl sulfate (IS), a protein-bound uremic toxin, is capable of inducing the release of IL-1β and other pro-inflammatory cytokines from macrophages with or without LPS exposure [55,56]. IS, which is implicated in cardiovascular disease [57], was able to activate NLRP3 in myotubes [34], suggesting that it may act as one of the circulating DAMPs in uremia.

## 9. Inflammation Up-Regulates Myostatin in Muscle of CKD5 Patients

Little information is available on the effects of TLR ligands on the synthesis of myokines in sepsis [26]. Myostatin (MSTN), a muscle-specific negative regulator of muscle mass, is up-regulated by LPS in C2C12 myoblasts, an effect antagonized by a specific TLR4 inhibitor [26]. In addition, MSTN deficiency prevented the increase in plasma macrophage inhibitory cytokine-1, as well as the reduction in muscle mass, while increasing survival in septic mice [58]. In muscle biopsies of a non-selected cohort of patients with CKD5 (including patients affected by wasting and inflammation), we observed an up-regulation of MSTN mRNA that was directly associated with IL-6 expression (see Ref. [59] for review). Zhang et al. were able to show that the up-regulation of muscle MSTN in patients with CKD depends on an IL6/Jak/Stat3/CAAT/enhancer-binding protein δ pathway, which is a potential target for therapy [17,60]. Sub-cutaneous injections of an anti-MSTN peptibody into mice with CKD suppressed circulating inflammatory cytokines and reversed the loss of body weight and muscle mass, suggesting that MSTN inhibition/antagonism is a new therapeutic opportunity [17,60].

## 10. Accelerated Cell Senescence in Uremic Muscle: A Novel Mechanism of Sterile Muscle Inflammation

Cellular senescence has emerged as an important driver of aging and CKD-related dysfunction in multiple organs, including muscle [61,62]. The term “cellular senescence” describes an irreversible growth arrest associated with morphological and functional changes, which include chromatin organization, gene transcription and protein secretion. Characteristics of senescent cells in vitro are an increased cell size, different sets of genes, including negative regulators of the cell cycle, the up-regulation of pro-survival pathways to resist apoptosis and the development of a senescence-associated secretory phenotype (SASP). The SASP is a distinctive secretome consisting of various pro-inflammatory cytokines, such as IL-1α, IL-1β and IL-6, and ligands of the Wnt–β-catenin pathway and TGF-β [62]. The repercussions of accelerated cell senescence are: (a) a loss of tissue repair capacity because of the cell cycle arrest; (b) accelerated fibrosis and dysfunction induced by the SASP [61]. The recent discovery that cell senescence is a driver for sterile muscle inflammation in patients with CKD is important [62], since it offers the possibility of using new senolytic/senostatics treatments.

CKD-associated wasting can overlap with aging-associated sarcopenia, therefore, expressing a combination of events occurring in aging and renal disease. Aging-associated sarcopenia is a loss of muscle mass and function in the elderly that reduces mobility, diminishes quality of life and can lead to fall-related injuries, which require hospitalization and extended rehabilitation [63]. A chronic low-grade inflammation state and a decrease in the autophagic process are key features of aging-associated sarcopenia [64]. Sarcopenia results from different mechanisms that include aging, physical inactivity, neuromuscular damage, anabolic resistance, lipotoxicity, oxidative stress, mitochondrial dysfunction and inflammation. At muscle protein levels, a loss of coordinated control between contractile, mitochondrial and sarcoplasmic reticulum protein is associated with post-translational modifications (see Ref. [64] for review).

## 11. Targeting Muscle Inflammation

Decreasing the production or removing circulating DAMPs in uremia and anti-inflammatory treatments that target the NLRP3 to IL-1 to IL-6 pathway of innate immunity may offer a new paradigm to treat CKD-related PEW and CVD. Besides the measure of circulating cytokines, the detection of serum levels of growth factors, such as VEGF-A, TGF-β1, FGF-21 and -23, proteases of MMP-2, -3, -9 and natural tissue inhibitors of TIMP-1 and -2, ox-LDL and isoprostane-8 and -15, has the potential to offer both early diagnosis and a follow up of target treatments.

### 11.1. Healthy Diets and Modulation of the Microbiome

Diets rich in vegetables, legumes, whole grains and fruits, with the avoidance of sugar-sweetened beverages, processed foods and trans-fats are recommended in prevention guidelines. The Mediterranean diet and plant-based diets can prevent the onset of age-related disorders, and has been associated with a long life [65]. Extra-virgin olive oil, which is contained in the Mediterranean diet, contains bioactive polyphenolic compounds that are known for their anti-oxidant and anti-inflammatory properties [66].

Toxins generated by the intestinal microbiome represent a potential therapeutic target. Small RCTs have shown that dietary fiber, sevelamer, syn-biotics, pre-biotics and anti-biotics may decrease blood levels of uremic toxins, including indoxyl sulfate, *p*-cresyl sulfate and *p*-cresol, as well as change the composition of some intestinal microbial species among patients with CKD and patients with ESKD. However, these effects are variable and results not consistent [67]. In animal CKD models, an oral adsorbent, AST-120, reduced inflammation and serum levels of indoxyl sulfate, and slowed kidney disease progression [68]. However, a large randomized, placebo-controlled trial in patients with CKD4 showed no significant effect of AST-120 on disease progression or death [69]. These conflicting results likely express the current gap in the understanding of the microbiome and its relationship to clinical outcomes. Nevertheless, the modulation of the microbiome with specific treatments or dietary interventions represents a promising research topic.

A decrease in circulating inflammatory biomarkers can also be obtained through smoking cessation, dietary interventions and physical exercise. In one study, weight loss in obese subjects decreased hsCRP by a similar magnitude, regardless of dietary composition [70]. In another study, dietary patterns with a high pro-inflammatory potential (processed meats, refined carbohydrates and sweetened beverages) were associated with higher CVD risk [71], which suggests that reducing the inflammatory potential of the diet may potentially provide an effective strategy for CVD prevention. In the PREDIMED study [72] involving persons at high cardiovascular risk, a Mediterranean diet supplemented with extra-virgin olive oil or nuts was associated with a lower incidence of major cardiovascular events than a reduced-fat diet.

### 11.2. Physical Exercise

Cardiovascular risk factors, cardiac autonomic control and left ventricular systolic function are ameliorated by physical exercise in ESKD patients [73,74]. Moreover, physical exercise has beneficial effects on chronic inflammation, muscle and bone strength in adults with CKD; improvements in inflammatory biomarkers can be accounted for through exercise-mediated shifts towards a less inflammatory immune cell profile, increased nitric oxide release and reduced monocyte infiltration into adipose tissue (see Ref. [75] for review). Among the treatment options for preventing the loss of muscle mass and function in ESKD patients, endurance or resistance exercises appeared to be the most useful. Additionally, expert opinion reports [76], position statements [77] and guidelines [78] have suggested that physical exercise needs to be considered as a standard of care treatment in patients with CKD. However, the available clinical evidence is mainly from small, short-duration (3–6 months) exercise intervention studies, and no conclusion could be reached for elderly ESKD patients [79,80]. In addition, major hurdles include the low percentage of patients who can actually perform physical exercise and a lack of resources to start an exercise program in dialysis units [80].

### 11.3. Targeting Cell Senescence

Senescence has been linked to the progression of muscle injury and age-related sarcopenia [81,82]. Recently, Huang et al. [61] observed that the administration of a senolytics cocktail to CKD mice for 8 weeks eliminated the disease-related elevation of senescence markers and depressed the high levels of SASP cytokines. Pharmacologically active compounds that manipulate cellular senescence have recently shown great promise in pre-clinical stages, and some of them are now being tested in clinical trials to reduce kidney fibrosis and chronic kidney damage. Senotherapeutic drugs induce the selective cell death of senescent cells (senolytics) or suppress markers of senescence (senomorphics), in particular the SASP. A number of agents with antiaging activity are currently under study. Another option to treat/prevent cell senescence is given by senostatics. Senostatics are drugs that “downregulate” the senescent phenotype without removing senescent cells. RAAS and the mineralocorticoid blockade, atorvastatin, rosiglitazone and omega-3 fatty acids are being re-examined for this purpose [83,84]. In addition, metformin and sirolimus (a mTOR inhibitor) have senostatic properties, since they both activate autophagy, improve mitochondrial function and can extend the lifespan. Other candidate drugs include inhibitors of IkB kinase, NF-κB and the Janus kinase pathway, which inhibit pro-inflammatory signaling pathways, disrupting SASP production. Recently, it has been observed that both the ketone body beta-hydroxybutyrate and sodium-glucose cotransporter-2 inhibition, whose action seems to increase the transcription factor Nrf2 anti-oxidant response, may down-regulate senescence [85,86].

Senomorphics can modulate the functions and morphology of senescent cells, or delay the progression of young cells to senescent cells. In general, senomorphics suppress SASP via targeting NF-κB, mTOR, IL-1a, p38 MAPK and other signaling pathways. Certain compounds have the dual capacity to function as both a senomorphic and a senolytic [87].

### 11.4. Nuclear-Factor-Erythroid-2-Related Factor 2 Agonists

Nuclear-factor-erythroid-2-related factor 2 (Nrf2) plays a strong anti-inflammatory role in many different tissues via the inhibition of the NF-κB signaling pathway. There is some evidence from experimental muscle atrophy models that Nrf2 agonists are protective against wasting. Male dystrophin-deficient (a model for Duchenne’s dystrophy) mice treated with the Nrf2 activator sulforaphane (SFN) underwent an increased expression of muscle heme oxygenase-1 and a decreased expression of NF-κB (p65) tumor necrosis factor-α, interleukin-1β and interleukin-6. In addition, the SFN treatment decreased the expression of NF-κB (p65). Collectively, these results showed that SFN-induced Nrf2 could alleviate muscle inflammation through inhibiting the NF-κB signaling pathway [88]. Recently, it has been also shown that cannabinoid CB_2_ receptor activation protects skeletal muscle due to ameliorating oxidative damage and promoting early skeletal muscle myogenesis, in part via Nrf2 signaling [89].

Nrf2 was shown to be down-regulated in the muscle of patients with CKD and in dialysis-treated ESKD patients [50,90], which suggests that it may serve as a potential therapeutic target. Among several Nrf2 agonists, bardoxolone methyl has been studied in large clinical trials [91,92]. In addition to bardoxolone, other Nrf2 agonists have been developed and are under evaluation in kidney and other organ diseases [93].

### 11.5. Targeting Inflammation to Treat Cardiovascular Disease in Patients with CKD

A current hypothesis is that controlling the inflammatory response through anti-cytokine therapies improves cardiovascular risk factors and, potentially, survival [93]. Given the number of levels in the regulation of the NLRP3 inflammasome, different sequences of its priming and activation steps can be targeted. Current treatments for NLRP3-related diseases include DAMP-inhibiting molecules, colchicine or biological agents that target TLR4, IL-6, IL-1β, IL-18 or their receptors [21,93]. In this regard, IL-1β inhibition has been shown to decrease the rate of adverse cardiovascular events in high-risk atherosclerosis patients with CKD [93]. Common concerns are related to long-term treatment toxicities, or to the physiologically protective role of inflammation as a defense mechanism against bacteria, viruses and fungi. In models of virus-mediated diseases, NLRP3-KO mice underwent more severe diseases than infected WT animals [94]. Therefore, studies have addressed the issue of safety, tolerability and feasibility, in addition to the efficacy of anti-cytokine therapies. In addition, several new molecules are being tested. The issue of anti-cytokine therapy to treat cardiovascular complications has been addressed by recent reviews [93,95].

### 11.6. Targeting Inflammation to Treat PEW

Pre-clinical studies suggest that blocking IL-1 signaling may be a new, promising treatment for CKD-associated muscle wasting. Anakinra blunts both IL-1α and IL-1β signaling through the IL-1 receptor [96]. The sub-cutaneous administration of Anakinra normalized muscle function in a mouse model of Duchene muscular dystrophy, an X-linked muscle disease characterized by muscle inflammation that is associated with increased circulating serum levels of IL-1β [97]. A 4-week treatment with Anakinra was shown to be safe in ESKD patients, and also significantly reduced blood CRP and IL-6 levels, but its effect on nutrition and muscle wasting has not been studied [98]. Dember et al. [99] randomized 80 inflamed hemodialysis patients to placebo or Anakinra for 24 weeks, with an additional 24 weeks of post-treatment safety monitoring. A decrease in hsCRP by 41% in the Anakinra group and 6% in the placebo group (not statistically significant) was observed. Anakinra was well tolerated and did not cause infections or bone marrow toxicity. These promising safety data and potential efficacy provide support for conducting larger trials of IL-1 inhibition to improve outcome in ESKD patients.

Recently, Cheung et al. [100] evaluated the efficacy of Anakinra in a mouse model of CKD-associated cachexia. Anakinra reduced the serum concentration and muscle expression of IL-6, TNF-α and IL-1β in CKD mice. This was accompanied by a reduced or normalized expression of negative regulators of muscle mass (Atrogin-1, Murf-1 and Myostatin). In addition, Anakinra attenuated the expression of transcriptional regulators of adipose tissue browning in inguinal white adipose tissue (WAT) of WT/CKD mice relative to controls. These data suggest that the IL-1 receptor antagonism may represent a novel targeted treatment for treating inflammation, adipose tissue browning and muscle wasting in CKD.

TLR4 activation is another possible target for approaches, either dietary or pharmacological, to disrupt the feed-forward loop of inflammation and wasting in uremia. TAK-242 (resatorvid) is a small-molecule inhibitor of TLR4 signaling that selectively binds to TLR4 and interferes with interactions between TLR4 and its adaptor molecules [23,101]. We recently observed that resatorvid prevented the uremic serum-induced increase in the inflammatory response in myotubes [50]. Resatorvid was previously used with success to prevent muscle wasting induced by sepsis in mice [102]; however, in a study of patients with sepsis and shock or respiratory failure, TAK-242 was well tolerated, but failed to suppress cytokine levels [103]. In CKD, the therapeutic target challenge remains as the suppression cytokine levels, as there is no information in patients with a micro-inflammatory state.

## 12. Conclusions

Skeletal muscle is now regarded as a component of the innate immune system, with primary importance in the response to infection. Sterile inflammation in muscle has an important role in the pathogenesis of CKD-related PEW. The activation of both the canonical TLR4–NF-κB–IL-6 pathway and the NLRP3 inflammasome through the use of endogenous ligands represents an important driver of muscle inflammation in advanced-stage patients with CKD. Both pathways are an up-stream target for approaches, either dietary or pharmacological, to disrupt the feed-forward loop of inflammation and accelerated muscle catabolism in uremia. Decreasing the gut production (or increasing the removal) of circulating DAMPs in uremia and/or anti-inflammatory treatments that target the NLRP3 to IL-1 to IL-6 pathway of innate immunity may offer a new paradigm to treat CKD-related PEW and CVD.
